# Effect of electromyostimulation on intramyocellular lipids of the vastus lateralis in older adults: a randomized controlled trial

**DOI:** 10.1186/s12891-021-04456-6

**Published:** 2021-06-22

**Authors:** Maya Hioki, Nana Kanehira, Teruhiko Koike, Akira Saito, Hideyuki Takahashi, Kiyoshi Shimaoka, Hisataka Sakakibara, Yoshiharu Oshida, Hiroshi Akima

**Affiliations:** 1 Graduate School of Medicine, Nagoya University, 65 Tsurumai, Showa-ku, Nagoya, Aichi 466-8550 Japan; 2 Department of Health and Nutrition, Tokaigakuen University, 2-901 Nakahira, Tenpaku, Nagoya, Aichi 468-8514 Japan; 3 Research Center of Health, Physical Fitness & Sports, Nagoya University, 1 Furo, Chikusa-ku, Nagoya, Aichi 464-8601 Japan; 4 Center for Health and Sports Science, Kyushu Sangyo University, 2-3-1 Matsukadai, Higashi-ku, Fukuoka, Fukuoka 813-8503 Japan; 5 Japan Institute of Sports Sciences, 3-15-1 Nishigaoka, Kita-ku, Tokyo, 115-0056 Japan; 6 Department of Human Wellness, Tokaigakuen University, 21-233 Nishinohora, Ukigai, Miyoshi, Aichi 470-0207 Japan; 7 Ichinomiya Kenshin College of Nursing, 5-4-1 Jouganndoori, Ichinomiya, Aichi 491-0063 Japan

**Keywords:** Energy substrate metabolism, ^1^H-magnetic resonance spectroscopy, Skeletal muscle, Blood biochemistry, Aging

## Abstract

**Background:**

Excessive intramyocellular lipid (IMCL) accumulation is a primary cause of skeletal muscle insulin resistance, especially in older adults, and interventions that reduce IMCL contents are important to improve insulin sensitivity. Electromyostimulation (EMS)-induced changes in IMCL content in older adults remain unknown. The purpose of this study was to clarify the effects of a single bout of EMS on the IMCL content of the vastus lateralis muscle in older adults.

**Methods:**

Twenty-two physically active, non-obese older men and women were randomly assigned to an EMS intervention group (69.0 ± 5.2 years, *n* = 12) or a control group (68.4 ± 3.5 years, *n* = 10). EMS was applied to the vastus lateralis (7 s on and 7 s off) for 30 min; control participants sat quietly for 30 min. IMCL content within the vastus lateralis was quantified with ^1^H-magnetic resonance spectroscopy (*n* = 7 per group). Fasting plasma glucose and insulin values were determined from blood samples collected before and after the EMS intervention.

**Results:**

EMS induced a significant reduction in plasma glucose (93.1 ± 9.6 to 89.5 ± 9.1 mg/dL, *p* < 0.01), but not IMCL content (15.7 ± 15.7 to 15.8 ± 13.1 mmol/kg wet weight, *p* = 0.49) or insulin (5.4 ± 2.4 to 4.7 ± 2.7 μIU/mL, *p* = 0.18). In the control group, no changes in IMCL content in the vastus lateralis was observed after prolonged quiet sitting.

**Conclusion:**

EMS intervention for 30 min induces changes in plasma glucose, but no changes in IMCL content in older adults.

**Trial registration:**

University hospital Medical Information Network (UMIN) Center ID: UMIN000020126. Retrospectively registered on December 222,015. https://upload.umin.ac.jp/cgi-open-bin/ctr_e/ctr_view.cgi?recptno=R000023242

## Background

Intramyocellular lipids (IMCLs) are an important energy substrate for adenosine triphosphate (ATP) production in skeletal muscle, especially during lower intensity physical activity [1]. However, IMCL contents are present at higher levels in older adults compared to younger adults [2–4]. Excessive IMCL accumulation is a primary cause of skeletal muscle insulin resistance [5]. IMCL contents are partly metabolized to ceramide, diacylglycerol, and Protein kinase C nuclear factor kappa B (PKC NF-κB), all of which can inhibit the insulin receptor signaling pathway in skeletal muscle [6]. Indeed, studies have demonstrated that IMCL contents are correlated with insulin sensitivity in humans, and increased IMCL contents in insulin-resistant participants are observed [7–9]. Thus, interventions that reduce IMCL contents are important in older adults to improve insulin sensitivity.

IMCL contents can be influenced by diet [10], physical exercise [11], and physical activity [12] in young adults, suggesting that IMCL turnover in response to exercise or diet indicates dynamic flexibility. However, compared with younger adults, IMCL contents in older adults are approximately 2-fold higher at rest (before resistance exercise) and remain unchanged during the recovery period, indicating less flexibility in IMCL turnover [13]. According to a previous study [14], IMCL oxidation is reduced in older compared with younger adults during aerobic exercise at the same intensities and times. Furthermore, muscle volume, function, and metabolism decrease with aging [15]. Age-related muscle atrophy is markedly apparent in the quadriceps femoris (QF), which influences daily physical activities. Intramuscular adipose tissue accumulation is also preferentially observed along with QF muscle atrophy with aging [16]. Therefore, exercise may be necessary to reduce the IMCL content in the QF in older adults.

Electromyostimulation (EMS) of skeletal muscles induces involuntarily muscle contraction and enhances energy substrate utilization [17]. According to a study using positron emission tomography (PET) and H215O [18], single-bout EMS intervention over the vastus lateralis (VL) muscle for 12 min induces a change in the blood flow of muscle tissue. EMS-induced muscle activation may thus induce acute changes in IMCL metabolism. Thus, EMS is commonly used in clinical settings as rehabilitation after spinal cord injury, stroke, or physical inactivity to improve skeletal muscle morphology, muscle function, and metabolism. Levels of oxidative enzymes are increased following EMS training for a period [19]. According to recently pilot study, IMCL content in combined type I and type II fibers decreased slightly after EMS rehabilitation in the patient with rheumatoid arthritis (BMI, mean ± SD 32.4 ± 7.1 kg/m^2^). Molecular adaptations of skeletal muscle to bout EMS loading also occur in a very short time [20, 21]. The change in IMCL content after acute exercise has been measured using biopsy and ^1^H-magnetic resonance spectroscopy (^1^H-MRS).

The purpose of this study was to clarify the effects in older adults of a single bout of 30-min EMS of the VL muscle on the IMCL content of the VL using non-invasive ^1^H-MRS. We hypothesized that EMS induces a decrease in the IMCL content of the VL in older adults.

## Methods

### Participants

Participant recruitment was conducted from January to July 2013, and all experiments were performed from July 2013 to February 2014. Twenty-two physically active, non-obese, independently living older men and women were enrolled in this study and were randomly assigned to an EMS group (*n* = 12) or a control group (without EMS) (*n* = 10). Exclusion criteria were based on the following requirements: 1) heart disease (e.g., myocardial infarction, angina pectoris, cardiac insufficiency), 2) cerebrovascular disease (e.g., cerebral infarction, hemorrhage), 3) extreme hypertension (e.g., systolic blood pressure (BP) ≥180 mmHg, diastolic BP ≥110 mmHg), and 4) with magnetic device such cardiac pacemaker. The Participants responded the clinical histories by questionnaires. Of the three participants with type 2 diabetes, one was receiving mitiglinide calcium hydrate (EMS group), one glibenclamide and mitiglinide calcium hydrate/voglibose, and one metformin (control group). All participants informed about the nature, purpose, and risks of the experimental procedures before their written informed consent was obtained. This study was approved by the Ethics Committee of the Graduate School of Medicine, Nagoya University. This is conforming to the standards set by the Declaration of Helsinki. The study adheres to CONSORT guidelines.

### Experimental protocol

**Fig. 1:**
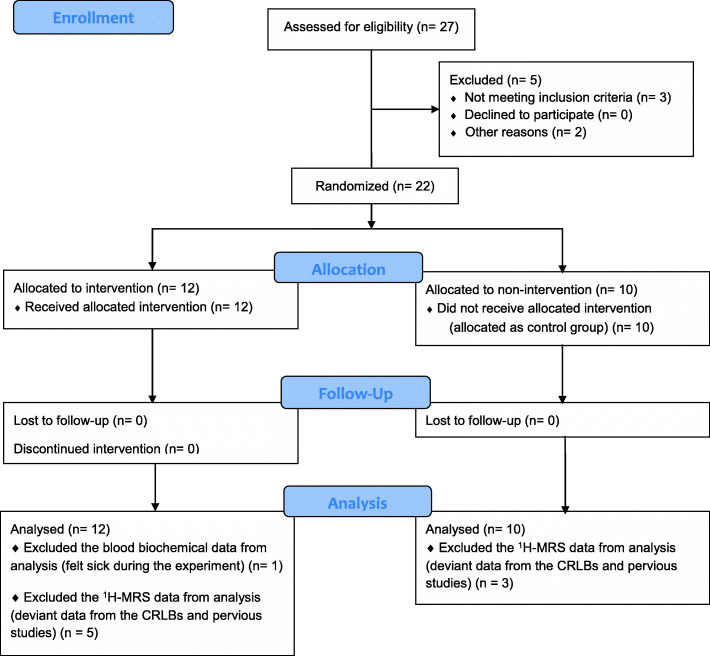
Flow Diagram illustrating the study. CRLBs, Cramer–Rao lower bounds

**Fig. 2:**
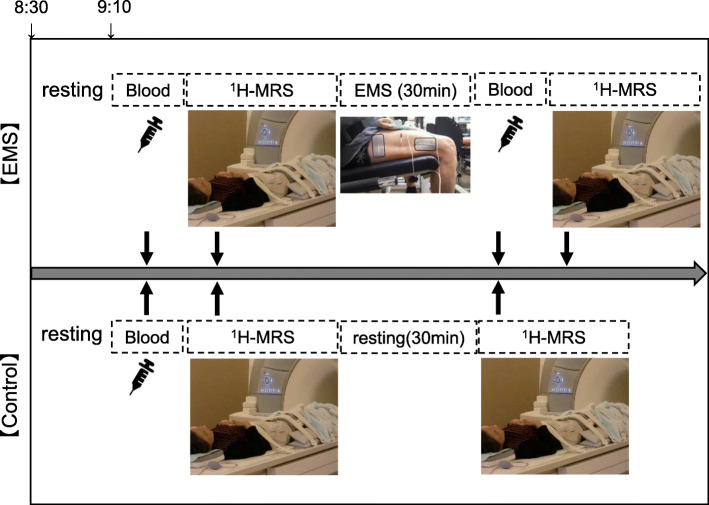
Experimental protocol of EMS (top) and control (bottom) groups. All participants arrived at the laboratory at 8:00 am after ≥10 h of fasting. We applied EMS to the VL of the right thigh for 30 min. IMCL and EMCL contents in the VL were quantified with ^1^H-MRS before and immediately after EMS in the EMS group, as well as before and after resting in the control group. Fasting blood samples were also collected before and immediately after EMS in the EMS group, as well as before resting in the control group. EMCL, extramyocellular lipid; EMS, electromyostimulation; ^1^H-MRS, magnetic resonance spectroscopy; IMCL, intramyocellular lipid; VL, vastus lateralis

Flowchart of the experimental procedures and participants is shown in Fig. 1. The experimental protocol for the EMS (top) and control (bottom) groups is shown in Fig. 2. Before beginning the study, all participants were familiarized with the EMS at the laboratory once a week × 3 times. The experimental protocol composed of measuring body composition and maximal voluntary contraction (MVC) during isometric knee extension, collecting blood samples, and performing assessment with ^1^H-MRS in the morning. For the ^1^H-MRS experiment, all participants arrived at the laboratory at 8:00 am after fasting ≥10 h. Fasting blood samples were collected before and immediately after EMS. We applied EMS to the VL of the right leg for 30 min. Whereas in the control group, blood was collected only before resting for 30 min, because, we predicted that change in biochemistry levels would not induce during resting for 30 min. The IMCL content in the VL was quantified by ^1^H-MRS before and immediately after the EMS in the EMS group, as well as before and after resting in the control group. Calculation of IMCL and extramyocellular lipid (EMCL) contents was performed with LCModel software (Stephen Provencher, Inc., Oakville, Ontario, Canada).

### EMS

Transcutaneous EMS (Trio 300, Ito Co., Ltd.) was performed on the VL. Voltage was delivered via two 5 × 9 cm electrodes (Axelgaard Mfg. Co., Ltd.) applied to the skin. One was placed approximately 5 cm distal to the greater trochanter over the VL, and the other was placed approximately 5 cm proximal to the superior aspect of the knee joint of the VL. EMS intensity was set until the individual’s maximum level of toleration. During EMS for 30 min, output muscle contraction was monitored with a custom-made dynamometer (Takei Scientific Instruments Co. Ltd., Tokyo, Japan), and the maximal value of EMS-induced muscle force was recorded. Stimulation was performed at a frequency of 30 Hz, pulse duration of 300 μs biphasic rectangular, and contraction/relaxation durations of 7 s on and 7 s off for 30 min.

Systolic BP, diastolic BP, and heart rate were measured before and after EMS for 30 min in the EMS group. Participants evaluated their pain with the visual analog scale on the day of the ^1^H-MRS experiment and 3 days after the experiment. The scale ranges from 0 to 100 mm with the zero-value indicating “no pain” and the 100-value indicating the “worst pain”.

### Blood collection and analysis

Blood tests were performed to determine the fasting levels of free fatty acid (FFA), triglyceride (TG), glucose, insulin, and hemoglobin A1c.

One participant felt sick during the experiment; this participant rested, consumed some sugar, and returned to the experiment. Therefore, we excluded the blood biochemical data after EMS for this one participant from analysis. Moreover, IMCL and EMCL data of this participant were not included.

### ^1^H-MRS

Before and immediately after EMS in the EMS group as well as before and after resting in the control group, ^1^H-MRS data were acquired on a 3.0-T MAGNETOM Verio (Siemens Healthcare GmbH, Eschborn, Germany) with a four-channel flex coil (366 × 174 mm) wrapped around the thigh. The voxel size was 11 × 11 × 20 mm. Visible adipose tissue, connective tissue, and vessels avoided, voxels were placed in the VL at the middle thigh between the greater trochanter and lateral condyle of the femur. In every participant, voxels were carefully placed at the same position while looking at the cross-sectional MR imaging at the mid-thigh level before and after EMS or resting in both groups. EMCLs are concentrated in distinct structures such as subcutaneous fat and fibrotic structures along muscle fibers in adipocytes (fasciae, septae). Depending on the exact position of the voxel, the EMCL signal can change by orders of magnitude [22].

Single voxel ^1^H-MRS measurements were performed using a point-resolved spatially localized spectroscopy (PRESS) sequence with the following acquisition parameters: TR/TE, 4000/30 ms, 128 averages. We were also used the unsuppressed water signal measured in the same voxel under the same shimming conditions as a reference signal [22].

### Post-processing

Fitting of all ^1^H-MRS data was performed using LCModel version 6.2-4A (Stephen Provencher, Inc.) [23]. From the scanners to a Linux workstation, data were transferred. Metabolite quantification was performed using water scaling and eddy current correction. The water concentration was postulated to be 42.4 mmol per kg wet weight based on the mean water content of 77% in muscle tissue [24]. The concentrations of IMCL-CH_2_ (1.3 ppm) and EMCL-CH_2_ (1.5 ppm) were computed as mmol per liter of muscle tissue (mM). IMCLs and EMCLs were collected for the T1 and T2 relaxation effects of the water reference using LCModel’s control parameter, atth2o, and determined using the following equation: ext. (−TE/T2) [1 − ext. (−TR/T1)] [25], assuming relaxation times T1 = 368 ms, T2 = 89.4 ms and T1 = 369 ms, T2 = 77.6 ms for the IMCL-CH_2_ and EMCL-CH_2_, respectively [26]. The concentration of total lipid content was calculated by summing IMCL-CH2 and EMCL-CH2 concentrations and dividing by 31. The value 31 was accorded to the assumption [22, 27] that the average number of methylene protons is 62 per triglyceride molecule (equivalent to 31 CH2 groups) [28]. To convert mM to mmol per kg wet weight, the value was divided by the tissue density (1.05 kg/L for normal muscle tissue).

^1^H-MRS spectra in participants in the EMS group (*n* = 12) and control group (*n* = 10) were acquired. All ^1^H-MRS data (*n* = 22) were processed using LCModel version 6.2-4A (Stephen Provencher, Inc., LCModel and LCMgui user’s manual) [23]. Before looking at the IMCL concentrations, the estimated standard deviations (SDs) (Cramer–Rao lower bounds [CRLBs]) expressed in percent of the estimated concentrations were checked. %SD < 20% has often been used as a very rough criterion to estimate acceptable reliability. Moreover, as error estimates of upper bounds, we compared our data with the data of previous studies (range of IMCL data, younger adults 2.4–26.3 mmol/kg wet weight; older adults 6.7–14.9 mmol/kg wet weight) [12, 29, 30], and obviously deviant data were excluded. In the present study, IMCL data (including before and after EMS) in four of the 12 older adults in the EMS group and IMCL data (including before and after resting) in three of the 10 older adults in the control group showed %SD > 20% according to CRLBs. Moreover, IMCL data in one of the 12 older adults in the EMS group showed obviously deviant data. Therefore, we excluded the data of these older adults from analysis and acquired IMCL data from the VL for seven participants in the EMS group and seven in the control group.

### Muscle strength

The participants were familiarized with the experiments at the laboratory at least 3 weeks before testing. MVC force during isometric knee extension were measured using a custom-made dynamometer (Takei Scientific Instruments Co. Ltd.). The dynamometer was strapped to the hip and thigh, and the knee joint was flexed at 90° (0° = fully extended). We adjusted the length of the vertical lever arm for the leg length of the participants and fixed with straps during MVC tests. MVC tests of the right leg were performed three or four at about 3-min intervals. We recorded the maximal attempt of two tests of three or four tests that yielded the highest force. Isometric knee extension force is expressed as an absolute value (Nm). During the EMS intervention, the EMS-induced isometric knee extension force was normalized to the muscle strength MVC.

### Physical activity levels

The physical activity level was acquired from records of a three-dimensional ambulatory accelerometer (Lifecorder, Suzuken Co., Nagoya, Japan) for 10 days. Technical details were provided by Kumahara [31] and the manufacturer. The values ranging from 0.06 to 1.94 g (1.00 g is equal to the acceleration of free fall) were assessed A 32-Hz sampling acceleration. The physical activity level was expressed as time and metabolic equivalent × hours (MET h). See our previous study [12] for additional details.

### Dietary parameters and analysis

Dietary intake during the 3 days before ^1^H-MRS assessments and the habitual dietary intake of the participants were determined by a nutritionist. During the 3 days before 1H-MRS assessments, the dietary intake was calculated from diaries and photos. Habitual dietary intake was estimated using a food frequency questionnaire, Ver. 2.0, which included 29 food and beverage items, and cooking methods of 10 series. The questionnaire asked about the average intake and frequency of consumption of each food. To describe consumption frequency, five categories were used as the follow; almost always, often, sometimes, rarely, or never. Dietary assessment showed the energy (kcal/body weight), carbohydrate (g/body weight), protein (g/body weight), and fat (g/body weight) intake for the 3 days before ^1^H-MRS as well as dietary habits.

### Statistics analysis

All data are shown as the means and SD. Analysis was performed by the investigators knowing which participants assigned to the EMS group. Differences between the EMS group and control group were analyzed using the Mann-Whitney test, and the differences between before and after EMS and resting for 30 min were analyzed using the Wilcoxon signed-rank test. The effect size (r) between the EMS and control groups was calculated by dividing Z by the square root of N (*r* = Z/√N), where N is the total number of participants, and the value of Z is calculated with the Wilcoxon signed-rank test. An effect size of 0.1 is considered small, 0.3 is moderate, and 0.5 is large. Analyses were performed with SPSS (version 24.0; SPSS Inc., Chicago, IL, USA). Statistical significance was set at *p* < 0.05.

## Results

### Characteristics of the study participants

The characteristics of the study subjects are provided in Table 1. The data for physical characteristics, blood biochemistry, skeletal muscle profiles, physical activity levels (min), dietary intake during the 3 days before ^1^H-MRS, and dietary habit profiles did not significantly differ between the EMS and control groups. Only the physical activity level (MET h) was significantly different between groups. Effect sizes (r) between EMS and control groups were small to medium.

**Table 1:** Characteristics of the study participants

	EMS	Control	Effect size (r)
No. of subjects (men/women)	12 (5/7)	10 (4/6)	
**Physical characteristics**			
Age (years)	69.0 ± 5.2	68.4 ± 3.5	−0.01
Height (cm)	159.0 ± 11.9	157.1 ± 6.9	−0.01
Weight (kg)	55.7 ± 10.6	56.0 ± 7.7	−0.06
BMI (kg/m^2^)	21.9 ± 2.3	22.7 ± 2.3	−0.11
**Blood biochemistry**			
Glucose (mg/dL)	94.1 ± 9.8	97.7 ± 25.4	−0.04
Insulin (μIU/mL)	5.5 ± 2.4	7.8 ± 7.3	−0.11
FFA (μEq/L)	714.0 ± 161.9	749.9 ± 265.3	−0.15
TG (mg/dL)	105.2 ± 77.4	87.2 ± 43.6	−0.08
HbA1c (%)	5.9 ± 0.4	6.0 ± 0.5	−0.13
**Skeletal muscle profiles**			
IMCL (mmol/kg wet weight)	15.7 ± 15.7	13.6 ± 15.8	−0.02
EMCL (mmol/kg wet weight)	28.5 ± 15.6	27.5 ± 14.2	−0.02
MVC during isometric knee extension (Nm)	71.6 ± 35.7	65.5 ± 25.0	−0.01
**Physical activity**			
Physical activity level (min)	93.8 ± 17.0	113.6 ± 27.2	−0.39
Physical activity level (MET h)	4.5 ± 1.0^*^	5.7 ± 1.7	−0.42
Number of steps	8991.8 ± 1722.7	11,123.2 ± 2990.0	−0.35
**Dietary intake during the 3 days before** ^**1**^**H-MRS**			
Energy (kcal/body weight)	35.8 ± 9.2	36.6 ± 7.3	−0.14
Carbohydrates (g/body weight)	4.9 ± 1.0	4.8 ± 0.9	−0.01
Protein (g/body weight)	1.4 ± 0.4	1.5 ± 0.4	−0.08
Fat (g/body weight)	1.1 ± 0.5	1.2 ± 0.3	−0.28
**Dietary habits**			
Energy (kcal/body weight)	36.8 ± 7.5	33.5 ± 7.6	−0.15
Carbohydrates (g/body weight)	5.0 ± 1.0	4.5 ± 0.9	−0.17
Protein (g/body weight)	1.4 ± 0.4	1.2 ± 0.3	−0.30
Fat (g/body weight)	1.2 ± 0.3	1.0 ± 0.3	−0.22

Values are means ± SD. ^*^p < 0.05 versus control group. *BMI* body mass index, *EMCL* extramyocellular lipid, *ES* effect size, *FFA* free fatty acid, *HbA1c* hemoglobin A1c, ^*1*^*H-MRS* magnetic resonance spectroscopy, *IMCL* intramyocellular lipid, *MET h* metabolic equivalent × hours, *MVC* maximal voluntary contraction, *TG* triglyceride. Data for IMCL and EMCL: EMS group, men *n* = 5, women *n* = 2; control group, men, *n* = 4, women *n* = 3

### EMS-induced force output

EMS-induced muscle force (N) was 37.1 ± 30.5 at the maximal value. This corresponded to 17.4 ± 11.3% of MVC. Systolic BP, diastolic BP, and heart rate did not change significantly in the EMS group. Pain evaluation with the visual analog scale was in the range of 0–25 mm (5.0 ± 11.2 mm) on the day of the ^1^H-MRS experiment and the 3-day period after the experiment. The mean visual analog scale score includes the data in the 5 out of 7 participants.

### IMCL and EMCL contents in the EMS and control groups

**Fig. 3:**
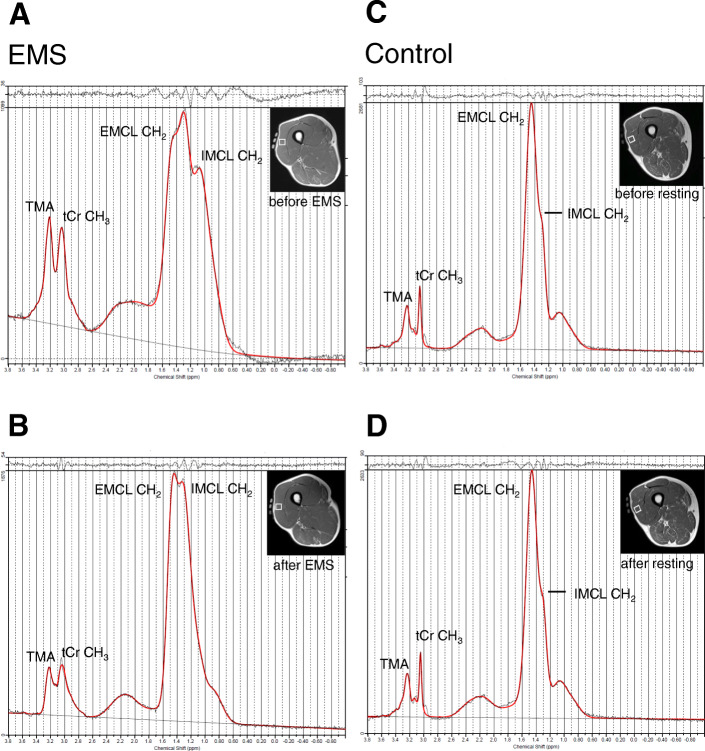
^1^H-MR spectra of the VL before and after the EMS intervention or resting. **A** and **B** before and after the EMS intervention (66-year-old men); **C** and **D** before and after resting for 30 min (66-year-old men). Signals are indicated as IMCL-CH_2_ at 1.3 ppm, EMCL-CH_2_ at 1.5 ppm, tCr-CH_3_ at 3.0 ppm, and TMA at 3.2 ppm. EMCL, extramyocellular lipid; IMCL, intramyocellular lipid; tCr, total creatine; TMA, trimethyl-ammonium

**Fig. 4:**
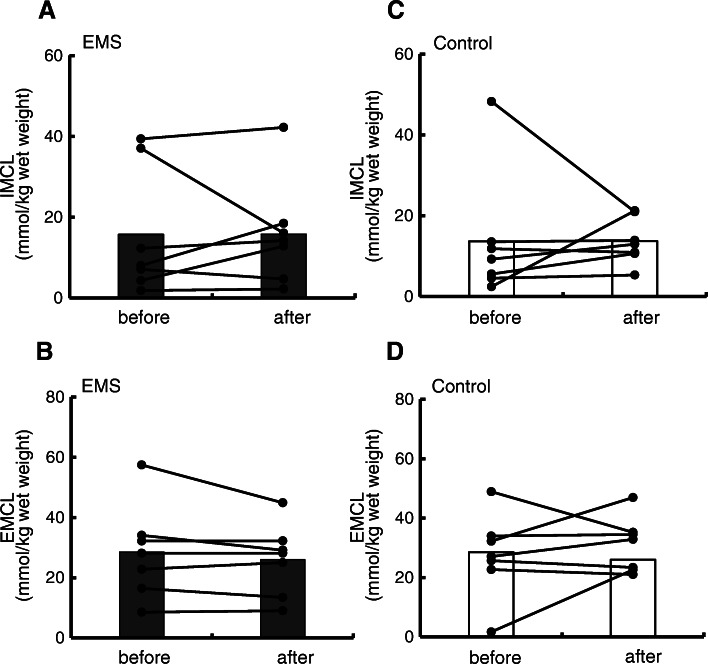
Comparison of IMCL and EMCL contents between before and after the EMS intervention or resting. EMS intervention (men *n* = 5; women *n* = 2, **A** and **B**) or resting for 30 min (men *n* = 4; women *n* = 3, **C** and **D**). EMCL, extramyocellular lipid; EMS, electromyostimulation; IMCL, intramyocellular lipid

^1^H-MRS spectra for the quantification of IMCLs obtained from the VL before and after EMS intervention or resting for 30 min for two 66-year-old men are shown in Fig. 3. Figure 4 shows the comparison of IMCL and EMCL contents between before and after the EMS intervention and resting for 30 min. No changes after EMS were observed in IMCL (15.7 ± 15.7 to 15.8 ± 13.1 mmol/kg wet weight, *p* = 0.49) or EMCL (28.5 ± 15.6 to 26.0 ± 12.0 mmol/kg wet weight, *p* = 0.49) contents of the VL. In the control group, no changes in IMCL (13.6 ± 15.8 to 13.7 ± 5.7 mmol/kg wet weight, *p* = 0.49) or EMCL (27.5 ± 14.2 to 30.9 ± 9.3 mmol/kg wet weight, *p* = 0.49) contents of the VL were observed after prolonged quiet sitting.

### Effect of EMS on the blood biochemistry concentrations

**Fig. 5:**
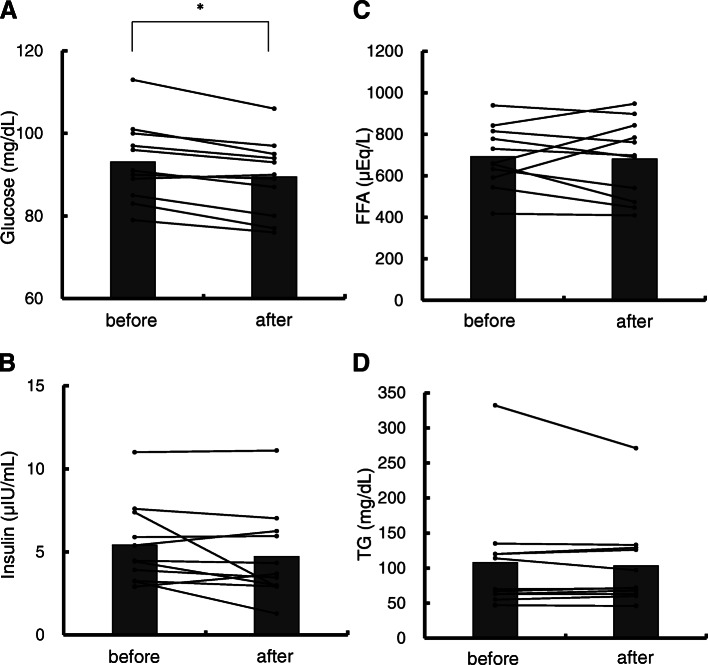
Comparison of blood biochemistry between before and after the EMS intervention. **A**, glucose; **B**, insulin; **C**, FFA; **D**, TG. men = 5; women = 6. *p < 0.01. EMS, electromyostimulation; FFA, free fatty acid; TG, triglyceride

Effects of EMS on the blood biochemistry concentrations are shown in Fig. 5. The plasma glucose concentration decreased significantly from before to after the EMS intervention (93.1 ± 9.6 to 89.5 ± 9.1 mg/dL, *p* < 0.01), whereas insulin (5.4 ± 2.4 to 4.7 ± 2.7 μIU/mL, *p* = 0.18), FFA (691.4 ± 148.5 to 681.1 ± 187.8 μEp/L, *p* = 0.72), and TG (107.8 ± 80.6 to 103.3 ± 63.5 mg/dL, *p* = 0.68) did not change significantly in the EMS group.

## Discussion

The present study demonstrated that EMS intervention for 30 min induces changes in plasma glucose, but no change in IMCL content in the VL of older adults.

EMS directly stimulates the motor nerve, thus resulting in a random pattern of recruitment; in other words, EMS-induced motor unit recruitment is non-selective (a reversal of the size principle) [17]. Indeed, molecular adaptations of skeletal muscle to EMS loading are drastic [20, 21]. Therefore, we speculated that EMS can induce a decrease in the IMCL content of the VL in older adults. However, our study results do not support this hypothesis. IMCL turnover in response to aerobic and resistance physical exercises is dynamic and flexible in young adults [32]. IMCLs are utilized as energy substrates by aerobic [33, 34] and resistance physical exercises [35, 36], and subsequently, FFAs are re-esterified in myocytes and recover. In contrast, IMCL turnover during physical exercise or physical activity in older adults seems to differ compared to that in younger adults. The contribution of IMCLs to whole-body fat oxidation during aerobic exercise is reduced with aging [14].

Our previous study also demonstrated that daily light-intensity physical activity is significantly and inversely correlated with IMCL content in younger adults, but not in older adults, suggesting that aging itself causes this effect [12]. Our results indicated that the IMCL content did not change after a 30-min EMS intervention in older adults. This result is agreement with a study showing that compared to younger adults, the IMCL content in older adults is approximately 2-fold higher at rest (before resistance exercise) and remains unchanged during the recovery period [13].

Inflexibility of IMCL metabolism seems to be induced by both a decrease in IMCL turnover and an increase in delivery of FFAs to muscle, which may be a result of a decline in muscle metabolic function with aging. Therefore, our finding suggests that IMCL metabolism during EMS is inflexible in older adults.

Our findings are consistent with the result in young adults, in whom whole body glucose uptake during a euglycemic clamp is acutely increased in response to 20 min of EMS as well as after EMS. These changes are insulin independent and AMP-activated protein kinase dependent [37]. Acute AMP-activated protein kinase activation suppresses glycogen synthesis and increases glucose transport [38]. We observed that plasma glucose decreased significantly after 30 min of EMS; in contrast, the insulin concentration was stable. During voluntary physical exercise, changes in fat and carbohydrate metabolism rates depend on the exercise intensity and duration. With increasing exercise intensity, plasma FFA oxidation progressively shifts to blood glucose oxidation. In contrast, during physical exercise, plasma FFAs and glucose availability progressively increase over time [39]. In contrast, according to a study using EMS, PET, and H215O, the percentages of activated regions of interest (i.e., those showing increases in the blood flow area) in the thigh area were 50.6% at 5% MVC and 62.2% at 10% MVC [18]. This indicates that EMS-induced knee extension force reflects an increase in involved muscle fibers. Therefore, we aimed to activate muscle contraction above 10% MVC during EMS. Indeed, the EMS-induced knee extension force showed 17% MVC. The VL volume accounts for approximately 30% of the QF muscles [40]. Our EMS intensity protocol may be high intensity for VL. Our results suggest that EMS can decrease plasma glucose in an insulin-independent manner.

EMS is an efficient to induce torque increases, however it is limited by the development of neuromuscular fatigue [41]. During maximal exercise, when a steady-state situation is never achieved, the ATP demand of muscle contraction is very high, and muscle fatigue occurs rapidly. Pulse frequency and duration of EMS give an impact on muscle torque production and fatigue [41, 42]. Although similar torque output during EMS, high-frequency EMS caused greater fatigue compared with low-frequency EMS [42]. Therefore, we selected the intermittent EMS (i.e., 7 s on-off cycle) at low-frequency stimulation (i.e., 30 Hz) to minimize the neuromuscular fatigue, however muscle fatigue might occur in the older adult by EMS in the study.

Many previous studies have reported that EMS training or rehabilitation result change in muscle morphology, function, and metabolism, i.e., oxidative enzyme activity, muscle fiber type, muscle fiber size, muscle strength [19]. Recently several studies [43, 44] have reported that IMCL content decreased slightly in the patient with rheumatoid arthritis after EMS rehabilitation for a period 16 weeks, and fasting glucose level decreased in the patients with type 2 diabetes after EMS rehabilitation for a period 8 weeks. Although our study verified change in bout of EMS, EMS rehabilitation for period might result influence of IMCL content.

EMCLs (i.e., fat tissue) are concentrated in distinct structures such as subcutaneous fat and fibrotic structures along muscle fibers in adipocytes (fasciae, septae) [22]. EMCLs are relatively metabolically inert, and capable of supplying a large fraction of metabolic substrates, mainly during very low intensity exercise [39, 45]. We found that EMCL content was constant after EMS in older adults. Therefore, our results suggest that lipolysis within EMCL (adipocytes) was not stimulated during EMS.

Our study has several limitations. First, the sample size for IMCL content was small. We instituted an exclusion criterion of %SD < 20% CRLBs as an estimate of acceptable reliability. Moreover, as error estimates of upper bounds, our data were comparable with the data of previous studies [12, 29], and obviously deviant data were excluded. The IMCL data did not satisfy the exclusion criterion in eight of the 22 older adults in both groups. In contrast, the effect size between EMS and control groups was small to medium. Second, although blood biochemistry was measured before and after EMS, blood biochemistry was measured only before resting for 30 min in the control group. One participant felt sick during the experiment. Therefore, we excluded the blood biochemical data after EMS of this one participant from analysis.

## Conclusions

In conclusion, the EMS intervention induced a significant reduction in plasma glucose, but not IMCL content, in the VL of older adults. The findings suggested that a single bout of 30-min EMS intervention affects plasma glucose, whereas IMCL metabolism during EMS is inflexible in older adults. Further studies are needed to determine whether the IMCL content decreases following EMS in older adults. For example, it is an experiment to determine the suitable protocol, i.e., EMS intensity, period, frequency duration time.

## Data Availability

The datasets used and/or analyzed during the current study are available from the corresponding author on reasonable request.

## Abbreviations

ATP: Adenosine triphosphate
BP: Blood pressure
CRLBs: Cramer–Rao lower bounds
EMCL: Extramyocellular lipid
EMS: Electromyostimulation
FFA: Free fatty acid
^1^H-MRS: Magnetic resonance spectroscopy
IMCL: Intramyocellular lipid
MET h: Metabolic equivalent × hours
MVC: Maximal voluntary contraction
PET: Positron emission tomography
QF: Quadriceps femoris
SD: Standard deviation
tCr: Total creatine
TG: Triglyceride
VL: Vastus lateralis
